# Supplementary material for: Polyketide synthase gene clusters in the ericoid mycorrhizal fungus Oidiodendron maius: genome-wide prediction, expression profiling, and functional characterization of knockout mutants
Source: Front Plant Sci. 2026 Sep 16;17:1924430. doi: 10.3389/fpls.2026.1924430 (PMC13623903; doi:10.3389/fpls.2026.1924430)
Supplement: Supplementary file 1 [file SupplementaryFile1.zip › Supplementary Materials/Supplementary Folder 1.zip!/Oidma1_AssemblyScaffolds_Repeatmasked/knownclusterblast/cluster4/jgi.p_Oidma1_15974_mibig_hits.html]

| MIBiG Protein | Description | MIBiG Cluster | MiBiG Product | % ID | % Coverage | BLAST Score | E-value |
| --- | --- | --- | --- | --- | --- | --- | --- |
| CCH26291.1 | putative\_O-methyltransferase | BGC0001158\_c2 | Bikaverin\_biosynthetic\_gene\_cluster | 33 | 81.0526315789 | 76 | 3e-14 |
| AAM70356.1 | CalO6 | BGC0000033 | Calicheamicin\_biosynthetic\_gene\_cluster | 32 | 93.6842105263 | 76 | 4e-14 |
| CCT67993.1 | bikaverin\_cluster-O-methyltransferase | BGC0000030 | Bikaverin\_biosynthetic\_gene\_cluster | 33 | 80.5263157895 | 75 | 8.9e-14 |
| ACS83775.1 | O-methyl\_transferase | BGC0000604 | GE2270\_biosynthetic\_gene\_cluster | 31 | 78.4210526316 | 67 | 1.4e-11 |
| AGY49596.1 | PbtM4 | BGC0001155 | GE2270\_biosynthetic\_gene\_cluster | 27 | 78.9473684211 | 63 | 3.5e-10 |
